# Dragging scientific publishing into the 21st century

**DOI:** 10.1186/s13059-014-0556-2

**Published:** 2014-12-11

**Authors:** Razib Khan, Laurie Goodman, David Mittelman

**Affiliations:** Department of Population Health and Reproduction, School of Veterinary Medicine, UC Davis, Davis, CA 95616 USA; GigaScience, BGI-Hong Kong Co. Ltd, 16 Dai Fu Street, Tai Po Industrial Estate, NT Hong Kong; Virginia Bioinformatics Institute and Department of Biological Sciences, Virginia Tech, Blacksburg, VA 24061 USA; Gene by Gene, Ltd, Houston, TX 77389 USA

## Abstract

Scientific publishers must shake off three centuries of publishing on paper and embrace 21st century technology to make scientific communication more intelligible, reproducible, engaging and rapidly available.

The Internet has massively disrupted how we communicate - primarily for the better. Many business sectors, however, have struggled to adapt to online platforms, with many simply resisting change. The newspaper industry is an example of a centuries-old industry persisting in the face of new conditions - until it can’t. In the early 1990s the Web began displacing traditional information delivery. By the mid 2000s it had become a widespread facet of life in many countries. Web 1.0 journalism translated ink to pixels, but as technology advanced the slow erosion of print became a landslide [1].

Scientific publishing is following a similar path, with its hesitance to adapt and slow (or no) adoption of the many advantages the Internet affords.

For now, scientific publishing remains profitable. Nevertheless, its sustainability rests upon antiquated pillars. Scholarly print journals date back hundreds of years to the availability of a cheap distribution method with the introduction of the printing press.

Most journals have made only incremental changes. A few have taken some advantage of the Internet and experimented with multimedia, but use of the medium has been limited primarily to extra content, such as unsearchable encyclopedic online supplements to accompany articles that maintain print page limits; or publishing many more articles by relaxing peer-review requirements for ‘novelty’, as exemplified by *PLoS ONE*, which has published 30,000 articles in 2013 alone [2]. Overall print-era anachronisms still persist through the continuation of page limits and surcharges and the release of discrete issues, as if all articles remain subject to print-only production schedules.

So how do we imagine the future of scientific publishing?

## Embracing the Internet to make science intelligible and reproducible

The scientific community has historically relied upon publishers for the advancement of science. The publishing community expanded its outlets as new methods of delivering content became available. But communicating science is more than just spreading information. How to realize this in 2014 is a fertile area for creative innovation as compared with 1650.

Some aspects of intelligibility are stylistic, while others are more substantive. On the substantive end, data and method release should be mandatory in a manner that enables rapid reproducibility on the part of the audience. Data storage costs have plummeted [3], so publishers could provide data hosting. Note: we are not talking about adding yet another repository, which many - rightly - feel are already so prevalent they make data more fragmented and less useable. However, there are growing challenges in properly curating data as data size grows. There is also a desperate need to organize the complex and growing amount of associated metadata, which is essential for intelligible data re-use and scientific reproducibility. This seems like an area in which publishers could take a major role. Having a database accessible and operable by a publisher will provide a ‘sandbox’, to organize, make available to reviewers, and build tools for data that are directly linked to specific publications. These data can then be distributed to appropriate community-approved data repositories, or publisher repositories can serve as short-term or (if needed) long-term means to host data types that have no community-approved repository.

Journals should also proactively engage researchers in developing and integrating best practices and standards, and incorporate tools by which the data associated with submitted work can be curated in a user-friendly comprehensible manner.

But reproducibility also requires the ability to manipulate data. Virtual environments like Arvados [4] hold promise for enabling reproducibility of data analysis with versioned scripts and tools. Authors can deposit into a virtual environment the data, tools and scripts they used to manipulate it, and end users can visit the virtual environment and operate on the data. The underlying data should be accessible enough that readers can manipulate analyses while viewing the paper, and see how robust the visualizations and statistics embedded are. One way to counter ‘*P*-value fishing’ is to make re-analysis so trivial that manipulations would be obvious. The substantive gains would not be limited to conventionally data-rich fields such as genomics. Imagine ‘wet lab’ publications where the article showcases a particular set of gels, but enables the viewing of all results inline through a gallery of alternative gel images. A publication should not be a static display of results and interpretations, but a distillation of the pith of the total scientific activity corresponding to a publishable unit.

An upside of radical transparency for scientists is that it increases the credibility of their work among peers. It is easier to persuade when your audience can see you put all your cards on the table. In computational fields, publishers can add further value by offering benchmarking resources to performance-test tools and methods using standard datasets and metrics. In genomics, performance tests and discussions exist on resources such as GCAT [5] and SEQAnswers [6], and these could be deployed through journal websites. Finally, another benefit of providing more in a publication is that modern technologies can give a sense of what the audience finds of interest. Which figures are being hovered over, which data are pulled, which tables are being rearranged?

Then there is style. Though today some papers arrive with multimedia and interactive features, most do not. Rather, they rely on the toolkit of tables and figures that dates back decades. A paper on the structural features of a biomolecule shouldn’t have figures of beautiful color plates; rather, one should be able to seamlessly view the structure from different angles. Mechanistic interactions should also be illustrated in animated form when necessary and possible. The Walter + Eliza Hall Institute of Medical Research in Australia has produced a set of animations of biological processes [7] that have amassed more than 1 billion views on YouTube. Clearly there is high demand for this sort of presentation. Several journals have already added or are in the process of adding some functionality to their once stagnant figures; for example, the JBC data viewer [8], which allows readers to manipulate imaging data. However, stronger support from the publishing houses, along with more innovation, is needed to provide the best possible reuse of all material in a publication.

## Publishers should facilitate engagement with science

In a sense, publishers will always be what they have always been: intermediaries between scientists keen to advance their field. The difference should be that publishers add value - modern value - rather than collecting rents through the control of historical legacies. Dedicated volunteers or individual institutions could perform all of the activities involved in creating a publication that incorporate information technology. However, those volunteers would be scientists who would have to take time out from their own primary activities.

There is now little preventing a researcher from setting up a weblog and releasing data and results directly. This is already being done and is exceedingly useful. But to create a publication house specializing in content delivery from the ground up requires time and resources that researchers don’t have, and without which it would be difficult to achieve the necessary level of professionalization, integration of presentation and substance of broad use to the community. There is a reason that we don’t live in an artisan DIY world: professionals add value.

There is an alternative path, where some publishers begin to offer services to their producers and a better product to their customers. They can make the publishing process easier, with generic format requirements, reducing the time commitment of researchers. Instead of just delivering content, journals can curate and personalize it, leveraging access to user data that only they have. Companies like Google can already create a researcher’s home page tailored to their interests. Journals have already incorporated systems that count and assess which articles and papers researchers tend to click, and could do so on a more individualized basis, allowing them to dynamically rearrange content to highlight those elements known to be of interest to a given user.

The use of threading in the ENCODE [9] publications provided a tool that allowed readers to generate articles on the fly that focused on content of particular interest to them. Setting this up was complex and time consuming but further investigation could allow the development of a tool that would allow such on-the-fly article building across entire publishing platforms, and better still across publisher boundaries. In addition to enabling rich, interactive data presentation, integration of papers across platforms, and methods that allow for easy reproducibility, the publishers could also begin to create policies and mechanisms that allow research to be presented closer to - if not in - real time, rather than having science move along at a publication-to-publication pace.

Cultural attitudes about how researchers share and how publishers interact with scientists remain a large hurdle, and yes, of course, these changes also require financial models. But as with the advent of open access, where most felt this wasn’t a monetarily prudent policy for publishers, innovation and creativity can go a long way toward reaching these goals. And as the self-declared communicators of science, publishers have a mandate to achieve this.

However, should publishers continue to do business-as-usual, then they will (and frankly should) become dinosaurs, while younger, more innovative and more robust communication venues take the lead. The publishing industry as it is constituted today is doomed to extinction. That isn’t an ‘if’. It’s a ‘when’.
